# High-Performance Washable PM_2.5_ Filter Fabricated with Laser-Induced Graphene

**DOI:** 10.3390/ma14195551

**Published:** 2021-09-24

**Authors:** Anh-Phan Nguyen, Won-Kyu Kang, Jung-Bae Lee, Jung-Bin In

**Affiliations:** 1 Department of Intelligent Energy and Industry, Chung-Ang University, Seoul 06974, Korea; anhpn@cau.ac.kr; 2 Soft Energy Systems and Laser Applications Laboratory, School of Mechanical Engineering, Chung-Ang University, Seoul 06974, Korea; zk273rvlb@cau.ac.kr (W.-K.K.); skzzjd@cau.ac.kr (J.-B.L.)

**Keywords:** laser-induced graphene, densification, flexible device, reusable, PM_2.5_ filter

## Abstract

This study demonstrates a novel application of laser-induced graphene (LIG) as a reusable conductive particulate matter (PM) filter. Four types of LIG-based filters were fabricated based on the laser-induced pyrolysis of thin polyimide (PI) sheets, each pyrolyzed on either a single side or both sides, with or without densification. The LIG filters exhibited a high removal efficiency while maintaining minimal pressure drop compared to a commercial fiberglass filter. The densified LIG (dLIG) filters displayed a higher PM_2.5_ removal efficiency (>99.86%) than regular LIG filters. The dLIG filters also exhibited excellent durability when tested for washability by ultrasonication in tap water. After being cleaned and left to dry, the structures of the dLIG filters were well-maintained; their filtration efficiencies were also well-maintained (less than a 7% change in PM_2.5_ removal efficiency), and their resistances only marginally increased (less than a 7% increase after five uses). These results demonstrate the robustness and reusability of the dLIG filters and the accessibility of their cleaning (not requiring aggressive cleaning agents). These promising features will enable the application of LIG in economical, scalable, and high-performance air cleaning.

## 1. Introduction

Air quality management and air pollutant filtration have been of significant interest to experts in various fields for mitigating negative environmental changes [1,2]. The adverse effects of air pollutants such as NO_X_, CO_X_, SO_2_, and ozone on public health are well-known. Particulate matter (PM), including fine dust particles (PM_2.5_), are hazardous due to their heavy metal compositions, especially cadmium, arsenic, lead, and zinc [3]. For instance, a PM of less than 10 µm (PM_10_) can settle in the lungs, and a PM of less than 2.5 µm (PM_2.5_) can penetrate blood vessels—they may have severe effects on the circulatory and respiratory systems [4,5]. Regarding the current COVID-19 pandemic, researchers have discovered that the coronavirus can survive for up to three hours in PM and can be directly carried into the cilium airway and lung if inhaled; an increase in PM_2.5_ density of only 1 µg/m^3^ could increase the COVID-19 mortality rate by 8% [6]. Thus, high-technology air filtration is essential for environmental damage control and suppressing the COVID-19 pandemic. 

Various types of fibrous filters have been developed for the filtration of PM in air. Among them, electret filters, which employ an electrostatic force generated by dielectric polymer fibers [7,8], are especially effective at PM collection. Nanofiber filters, made of highly polar polymers, have a high PM collection capability [9,10]. The hierarchical carbon nanotube (CNT)/quartz fiber filter exhibited a two-order-of-magnitude reduction in the penetration rate of sub-micron aerosols compared to conventional high-efficiency particulate air (HEPA) filters [11,12]. However, these non-conductive filters should be fabricated as a densely stacked multi-layer structure to achieve a high collection rate [13,14] and suffer from large product volume and a significant pressure drop. 

In contrast, high-conductivity filters exploit the Coulombic attraction between the charged particles and the charged dust collector (or electrostatic precipitators (ESPs)). This arrangement is markedly more efficient at PM collection than dielectric polar polymers because the Coulombic force can be amplified at a high bias voltage and has a broader range than the dielectrophoretic force generated from a permanent dipole of a polar polymer [15]. Therefore, the conductive filter can reach a high collection rate while experiencing minimal pressure drop [16,17]. 

With its outstanding electrical, mechanical, and chemical properties, graphene is a promising material in ESPs for PM collection. However, its fabrication and assembly into a filter are expensive, inhibiting its practical use as a filter material. Recently, laser-induced graphene (LIG) has been suggested as a promising functional nanomaterial alternative to conventional graphene. LIG is produced by irradiating a polymer, such as polyimide (PI), with a near-UV-to-IR laser [18,19] even under ambient conditions. Moreover, although its lattice structure is not as ideal as that of conventional graphene, LIG still exhibits the valuable properties of graphene. LIG is mechanically flexible, electrically conductive, and highly porous [20,21]. In this respect, LIG is a promising filter material that implements the high filtration performance of conventional graphene at a reasonable cost [22,23,24]. 

Nonetheless, several technical challenges must be overcome for the practical use of LIG for PM filtration. Although LIG is inherently porous and thereby permeable to liquid and gas, when an LIG film is generated based on a polymer sheet, the fluid flow to the thickness (or normal) direction is frustrated by the underlying polymer. A freestanding LIG sheet can be produced using a thin polymer sheet or by irradiating both sides of a polymer sheet with a laser. However, as-produced LIG is generally fragile; the graphene flakes in LIG do not strongly bind together and its porous structure tends to break apart when externally disturbed. Consequently, when subjected to a PM flow over time, LIG can disintegrate from the filter. 

The fragile structure of LIG also removes the washability of LIG filters. The washability and reuse of filters are crucial for their economic and eco-friendly use. Various reusable filters have been developed that can be cleaned mechanically, such as by pulse jet processing [25] or chemically with a polar substance [26] such as DI water, ethanol, or ethylene glycol [27]. However, the structure of as-produced LIG is insufficiently robust to endure external agitation for cleaning. In summary, LIG is a promising conductive filter material for efficient air filtration. However, for its practical use, the fabrication of mechanically reinforced LIG filters in a thoroughly permeable form is critically required. 

This study aims at improving the mechanical robustness of LIG for reuse and fabricating LIG-based filters in a simple and scalable manner. A high-performance washable air filter was developed based on densified LIG (dLIG). As periodic laser beam pulses were applied to a PI sheet, LIG was generated and simultaneously perforated with no need for additional machining. LIG could be produced on both sides of the PI sheet to improve the collection rate, and its structure was reinforced by densification. The as-produced LIG was densified using the duplicate laser pyrolysis method [28,29]. Various types of LIG filters were fabricated based on these methods and tested to compare their PM filtration performance. dLIG exhibits strong bonding between LIG flakes and the substrate, resulting in excellent washability, superior to regular LIG. 

## 2. Materials and Methods

### 2.1. Materials and Laser Irradiation

Commercially available PI films (Kapton^®^ HN Polyimide Film, ~50 μm thick, tensile strength: 231 MPa, McMaster-Carr Supply Company, Elmhurst, IL, USA) were used as a precursor for sLIG, which is a conventional form of LIG. The surfaces of the PI films were cleaned with a mixture of ethanol and water prior to laser irradiation. The cleaned PI film was raster-scanned using a CO_2_ laser engraver (C40–60 W, wavelength: 10.6 μm, beam diameter: ~260 μm, Coryart, Anyang, South Korea). An optical chopper (MC2000B-EC, Thorlabs Inc., Newton, NJ, USA) with a 40% duty-cycle chopper wheel (MC1F10A, Thorlabs Inc., Newton, NJ, USA) was used to modulate the laser beam output. The rotational speed of the optical chopper was adjusted to produce 1-kHz laser beam pulses. The laser irradiation area or filter area was 1.5 × 1.5 cm^2^. 

For the fabrication of dLIG, the sLIG surface was coated with an additional PI layer. The sLIG surface was coated with a poly (pyromellitic dianhydride-co-4,40-oxydianiline) amic acid (PAA; 12.8 wt% in 80% NMP and 20% aromatic hydrocarbon; Sigma-Aldrich, Burlington, MA, USA) solution and left to rest for ~10 min until the PAA was sufficiently absorbed into the porous sLIG. A spin coater (SPIN 1200D, MIDAS SYSTEM CO. LTD., Daejeon, Korea) was used at 1000 rpm for 30 s, 1500 rpm for 20 s, and 500 rpm for 20 s to remove the excess PAA solution [30]. For the complete removal of solvent, the PAA-coated LIG was preheated on a hot plate at 100 °C for 20–30 min and placed in a vacuum oven (VDO27, Coretech, Hwaseong, Korea) at 100 °C for one hour. The sample was then transferred to a hotplate and subsequently annealed at 250 °C for 30 min for thermal imidization, resulting in the formation of PI from PAA.

### 2.2. Characterization

Short strips of copper tape were connected to two parallel sides of a square sample, covering each side entirely to measure the electrical resistance of an LIG sample. The sample resistance was then characterized with a digital multimeter (34450A, Keysight Technologies, Santa Rosa, CA, USA) by measuring the resistance between the two copper tape strips. The pressure drop in the LIG filters was measured using a pressure-and-flow meter (DT-8920, CEM CO. LTD., West Bengal, India). Scanning electron microscopy (SEM) (S-3400N, Hitachi LTD., Tokyo, Japan) and field-emission SEM (FE-SEM) (SIGMA, Carl Zeiss AG, Oberkochen, Germany) were used to capture images of the micro-structures of LIG samples. 

### 2.3. PM Generation and Filtration

PM particles were generated by burning commercial incense (Lotte Prayer Incense Sticks, Lotte Aluminum Co., Ltd., Seoul, Korea). The concentration of as-produced PM particles was lowered by mixing the particle flow with a controlled airflow. Testing revealed the composition of the generated PM, categorized into PM_1_, PM_2.5_, PM_4_, and PM_10_, as shown in Figure S1 in Supplementary Information. The concentration of particles (PM_1_, PM_2.5_, PM_4_, and PM_10_) was measured using a PM counter (SPS30, sensor limit: 1000 µg/m^3^, Sensirion Korea CO. LTD., Gyeonggi-do, Korea). An ionizer (TFB-YD12R, highest voltage output: −15 kV, ion concentration: 200 × 10^6^ pcs/cm^3^, TRUMPXP Electronic Technology CO. LTD., Anhui, China) was used to charge the PM with negative charges. A commercial fiber-type filter (Hyundai Micro GLASS Fiber Filter, GF-A, Hyundai Micro CO. LTD., Seoul, Korea), which mechanically filtrates PM, was used as a control for the performance experiment. 

## 3. Results and Discussion

### 3.1. Fabrication of LIG Filters

**Figure 1:**
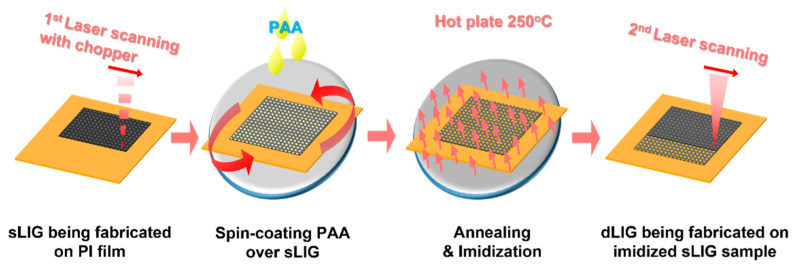
Fabrication process of LIG PM filters.

Figure 1 illustrates the fabrication process for LIG filters. A PI film was irradiated with a CO_2_ laser beam at a power of 30 W and a scan speed of 200 mm/s with 150 μm spacing between scanning line paths. As the laser beam output was modulated by an optical chopper, periodic hole arrays were produced through the PI film. Consequently, LIG (or sLIG) was generated in the neighboring areas of the holes, covering the entire top area of the PI film. This filter is hereafter denoted as single-faced sLIG (SF-sLIG). For the fabrication of double-faced sLIG filters, denoted as DF-sLIG, the same laser processing was applied to the back side of the sLIG-on-PI film with the same process parameters but at a different laser power of 5.2 W and with different spacing between the scanning line paths (125 μm). The power was lowered to obtain a stable freestanding filter membrane, where a thin PI layer intermediated between both LIG layers. 

**Figure 2:**
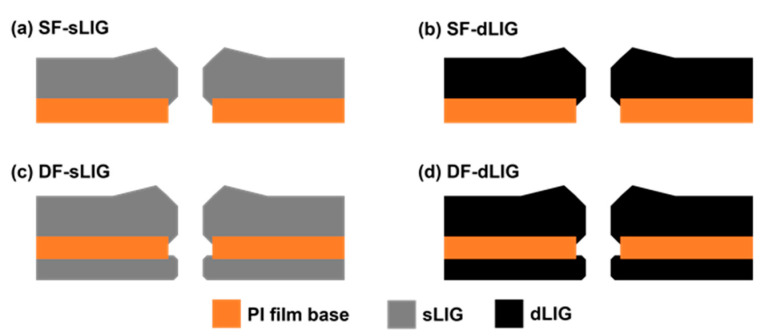
Cross-sectional schematics of different LIG filter types: (**a**) SF-sLIG, (**b**) SF-dLIG, (**c**) DF-sLIG, and (**d**) DF-dLIG. The different shades (gray and black) signify different densities of the generated LIG.

For fabricating a dLIG filter from an sLIG sample, the sLIG surface was coated with PI as described in Section 2.1. The PI-coated sLIG sample was irradiated again with a laser at 4.8 W, where the additional PI layer was sufficiently carbonized into LIG [28]. Above this power, severe ablation could occur, and laser-induced pyrolysis tended to be unstable. Scanning with a laser was performed at a speed of 200 mm/s with a 125-micrometer gap between the scanning lines. Consequently, dLIG was generated from the sLIG layer. For fabricating DF-dLIG filters, the same process was applied to both sides of a DF-sLIG sample. Figure 2 summarizes the cross-sectional structures of the SF-sLIG, SF-dLIG, DF-sLIG, and DF-dLIG. 

### 3.2. Morphology of LIG Filters

**Figure 3:**
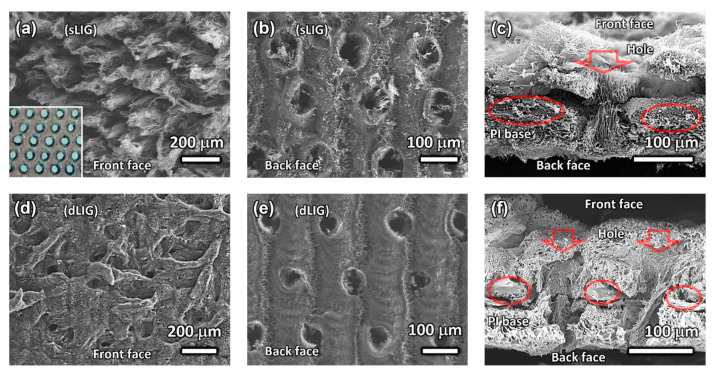
SEM images of different views of different LIG filters. Top-down view on (**a**) the front side of the SF-sLIG filter, (**b**) the back side of the DF-sLIG filter, (**d**) the front side of the SF-dLIG filter, and (**e**) the back side of the DF-dLIG filter. Cross-sectional view of (**c**) the DF-sLIG filter and (**f**) the DF-dLIG filter; the red arrows denote holes with corresponding size; the ovals denote traces of remaining PI film base.

The morphologies of various LIG filters were investigated using SEM (Figure 3). Figure 3a illustrates the top-view SEM image of the front side of the SF-sLIG. Distinct fibrous LIG structures protruded from the PI substrate. This fibrous LIG was observed by Duy et al. [31] and named LIG fibers (LIGFs). In our study, the front side of the PI sheet was irradiated with a CO_2_ laser at high power (30 W, or 56.5 kW/cm^2^ in terms of irradiance). Consequently, laser ablation occurred from the center of the beam spot, producing a hole through the PI sheet and LIGFs on the hole’s rim.

The hole structure was not observable from the top-view SEM, being shrouded by the relatively tall LIGFs. However, through-hole arrays produced by the periodic laser pulses were unveiled after the LIG was entirely removed by ultrasonication in water (presented in the optical microscopy image; inset of Figure 3a). In contrast, as depicted in Figure 3b, the LIG generated on the back side was relatively flat because the back side PI was continuously irradiated with the laser at a relatively low laser power (5.2 W) and a tight scanning gap (125 µm), unambiguously revealing the through-holes previously produced by the laser irradiation at the front side. 

Figure 3c illustrates the cross-section of a hole in the DF-sLIG filter. Although both sides of a PI sheet were irradiated with a laser, the PI part not affected by the laser was present between the LIG layers, supporting the generated LIG. The remaining PI was strong enough for the LIG filter to be used as a freestanding membrane subjected to a pressure difference. However, the LIG–PI adhesion was weak with evident delamination (Figure 3c) when the cross-section sample was prepared by tearing the filter film. 

The dLIG morphology produced on the front side of the DF-dLIG differed markedly from that of the sLIG. Figure 3d illustrates the top-view SEM image and reveals the noticeably denser structure of the dLIG compared to that of the sLIG (Figure 3a). Coarse LIGF structures were not visible because the LIGFs became wet with the PAA solution and collapsed by spin-coating during densification. The morphology also suggests that the collapsed LIGFs were bound to the dLIG surface, partially covering the holes’ openings. The sLIG impregnated with PAA was converted into additional LIG upon duplicate laser irradiation, densifying sLIG into dLIG. The laser power used was low (4.8 W), and no additional LIGFs were produced. The SEM image captured from the back side of the DF-dLIG (Figure 3e) reveals that the hole size is noticeably smaller than that of the DF-sLIG (Figure 3b), possibly because of the additional generation of LIG by densification. 

A cross-sectional view of holes in the DF-dLIG filter (Figure 3f) illustrates the laser-drilled holes and supporting PI structure. However, in contrast to the sLIG, the adhesion of the dLIG to the supporting PI was starkly stronger, suggesting the structural robustness of DF-dLIG filters in the application of PM filtration. The average thicknesses of the SF-sLIG, SF-dLIG, DF-sLIG, and DF-dLIG filters were approximately 144, 111, 184, and 144 μm, respectively. A significant reduction in thickness was found by densification. This decrease predominantly reflects the change in the LIGF structure on the front side: the collapse of the LIGFs, resulting in a decrease in the LIG extrusion height from the substrate. 

### 3.3. Filtration Performance

**Figure 4:**
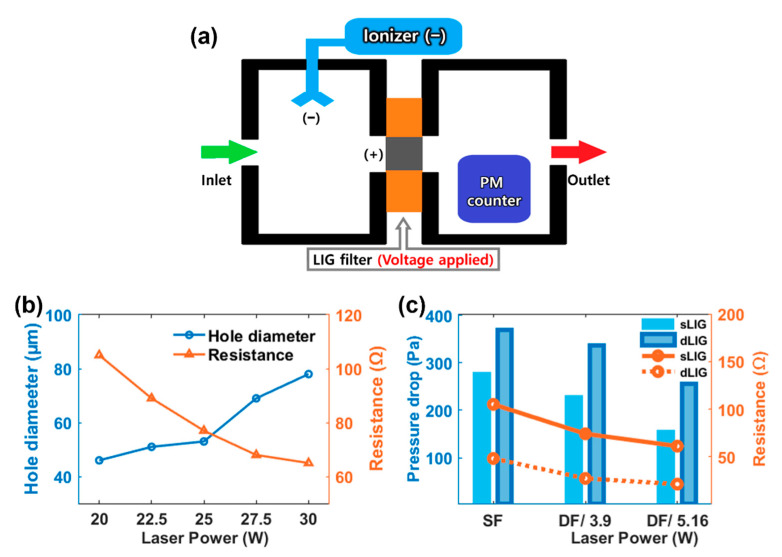
(**a**) Schematic of the air filtration setup. The PM counter sensor is placed in the right side of the chamber, while the PM (incense) is introduced into the left side of the chamber. The LIG filter is sandwiched between the two chambers; (**b**) Hole size and electrical resistance of sLIG filter samples drilled with different laser powers; (**c**) Pressure differences and resistances of single-faced (SF) and double-faced (DF) LIG filters fabricated with different back-side laser scanning powers: 3.9 and 5.2 W.

The performance of the LIG filters was evaluated using a home-built air filtration setup that consisted of two chambers: one upstream and one downstream (Figure 4a). A digital photograph of the setup is available in Supporting Information (Figure S3). These chambers were connected via an LIG filter to be tested. O-rings were used for an air-tight connection. Diluted incense smoke was continuously introduced to the upstream chamber using an air compressor. The concentration was adjusted close to the measuring limit of the PM counter (1000 µg/m^3^). While the smoke particles were negatively charged by the ionizer placed in the upstream chamber, a positive potential of +12 V was applied to the LIG filter. The air with the charged particles flowed through the filter. The negatively charged PM was collected at the positively charged filter via the Coulombic interaction. The PM concentration and distribution by sizes inside the downstream chamber were obtained using the PM counter. The filtered air finally escaped from the downstream chamber via the outlet open to ambient air. 

Through-holes of different sizes were produced at different laser powers; the electrical resistances of the sLIG also changed with the laser power (Figure 4b). The hole diameter increased with laser power, but the resistance inversely decreased because the amount of produced LIG increased with the laser power. However, at a laser power above 30 W, the LIG sample was severely damaged and deformed. A large hole is favorable for reducing the pressure drop, and a lower resistance is required to maintain a high PM collection rate. Therefore, the sLIG was produced at 30 W to fabricate the LIG filters. The macro holes served as primary passages for airflow and as highly conductive sites with fibrous LIG structures. As the hole sizes are much larger than those of the PM to be collected, enlarged hole sizes could be detrimental to filtration performance. However, as discussed below, the filtration performance of the perforated LIG filters was satisfactory because of the long-range Coulombic attraction. 

Figure 4c illustrates the resistances and pressure drops of the SF-sLIG and SF-dLIG filters. The DF-sLIG and DF-dLIG filters were produced at laser powers of 3.9 and 5.2 W for back side pyrolysis. When the laser power was above 5.2 W, the sample structure collapsed and could not be used as a filter. The result reveals that the electrical resistances of the dLIG samples were lower than those of the corresponding sLIG samples in all the cases, consistent with the findings of previous studies [28,32] because of the increased density of the dLIG, increasing conduction paths within a given volume. For all types of LIG filters, the resistance decreased as the laser power increased. 

The pressure drop across an LIG filter was measured using the filtration setup at a flow rate of 6.4 L/min. As depicted in Figure 4c, the pressure drop for DF filters was smaller than for their counterpart SF filters because the hole size was widened by the additional laser-induced pyrolysis on the back side of the PI. In all the cases, the dLIG filters exhibited larger pressure drops than the sLIG filters, attributed to the decreased hole size caused by the additional coating with PAA for densification. Nevertheless, the pressure drop of DF-dLIG filters was negligible (e.g., ~255 Pa or 0.25% of atmospheric pressure). For optimum performance in the subsequent filtrations, all the DF-LIG samples were fabricated at a laser power of 5.2 W laser for back side pyrolysis.

**Figure 5:**
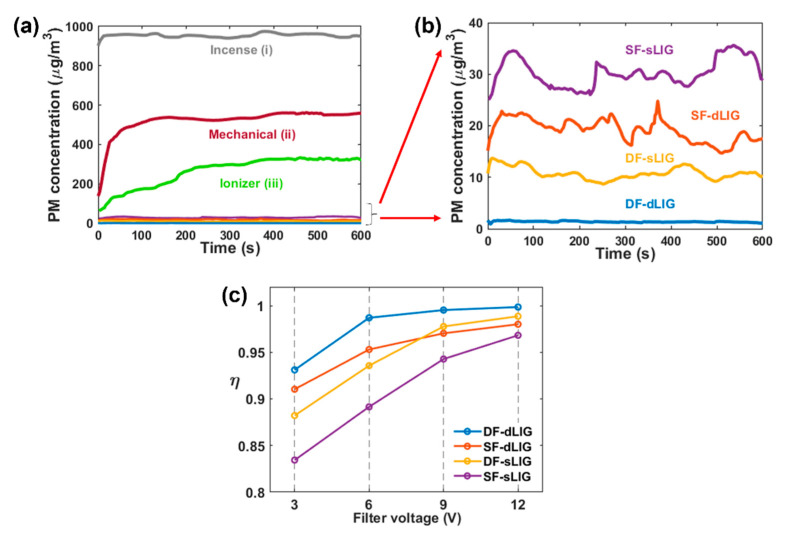
Results of PM_2.5_ removal testing. (**a**) PM_2.5_ concentrations measured in the downstream chamber for several cases: (**i**) diluted incense flow without filter, (**ii**) unbiased DF-dLIG filter (0 V) with ionizer off (0 V), (**iii**) unbiased DF-dLIG filter with ionizer activated; (**b**) PM_2.5_ concentrations for biased (12 V) SF-sLIG, SF-dLIG, DF-sLIG, and DF-dLIG filters with ionizer on (enlarged part of (**a**) in the range 0–40 µg/m^3^); (**c**) PM_2.5_ removal efficiency of different filters with different voltages applied to LIG.

Figure 5a,b illustrate the filtration performance of the various LIG filters for PM_2.5_. The filter efficiency was determined based on the following equation:

(1) $$\eta =1-\frac{C_{out}}{C_{in}},$$

where *C_in_* and *C_out_* are the particle concentration (µg/m^3^) upstream and downstream of the filter, respectively. Figure 5a illustrates the real-time concentration data measured from the PM sensor in the downstream chamber for several cases. Case (i) corresponds to the diluted incense flow with no filter installed. The average concentration of PM_2.5_ was ~952.99 µm/m^3^; this value is considered to be *C_in_*. For Case (ii), pure mechanical filtering was examined by adopting unbiased (0 V) DF-dLIG without using the ionizer under the same diluted incense flow; its efficiency was ~45.05%. When the ionizer was activated in Case (iii), the efficiency was raised by 27.51% (to 72.56%) but was still was less than 80%.

For all types of LIG filters (SF-sLIG, SF-dLIG, DF-sLIG, and DF-dLIG), when PM ionization and the bias voltage to filter (+12 V) were all activated, the PM concentration decreased dramatically, resulting in efficiencies above 96% (Figure 5b). These results reveal an important cooperative effect of PM ionization and filter biasing. Among the different filters, the efficiency was highest for DF-dLIG (η: 99.86%; post-filtration PM_2.5_ concentration: 1.2 µg/m^3^), and the efficiency of various LIG filters decreased in the order of DF-dLIG > DF-sLIG > SF-dLIG > SF-sLIG (Table 1). The DF configuration improved the filter efficiency, possibly due to the increased surface area and low electrical resistance. The various LIG filters could remove PMs of various sizes (PM_1_, PM_2.5_, PM_4_, and PM_10_), and their efficiencies at 12 V bias voltage were all above 96% (Figure S2 in Supporting Information). For every filter, the PM removal of PM_1_ was higher than that of any other type of measured PM. 

Figure 5c illustrates the efficiencies of DF-dLIG, SF-dLIG, DF-sLIG, and DF-sLIG for PM_2.5_ obtained at different bias voltages (3, 6, 9, and 12 V). The efficiency increased with the bias voltage due to the pronounced Coulombic attraction at high voltages. The changes in efficiency with bias voltage suggest that the efficiency improvement at increased voltages tended to reach a plateau relatively earlier at 6 V for the dLIG filters compared to 9 V for the sLIG filters. This early saturation can be attributed to the lower electrical resistance of the dLIG filters compared with the sLIG filters (Figure 4c). The DF filters’ efficiencies were higher than those of their counterpart SF filters because of the contribution of the additional LIG placed on the back side. Consequently, the efficiency of the DF-dLIG filter was highest among those of the examined LIG filters for all voltages. 

The DF-dLIG pressure drop was higher than that of the DF-sLIG but still negligible at only 19% that of the commercial filter. Nevertheless, the PM_2.5_ penetration rate (the rate of PM passing through the filter without being collected, defined as 1 − η) for the DF-dLIG filter was only 0.14%. In contrast, the conventional filter had a 2.53% PM_2.5_ penetration rate. The quality factor (**QF**) is a widely used metric that illustrates the relationship between the pressure drop and the efficiency of a filter, as depicted in the following equation:

(2) $$QF=-\frac{\ln\left(1-\eta \right)}{△P}\;;$$

high **QF** values imply a high filtration performance. All the LIG filters exhibited high **QF** values superior to the commercial filters. 

Table 1 summarizes the efficiencies, pressure drops, and **QFs** of the LIG filters and the commercial (micro glass fiber) filter, evaluated based on PM 2.5 filtration with 12 V bias voltage. All the types of LIG filters vastly outperformed the commercial fiberglass filter in terms of pressure drop and **QF**. All the LIG filters other than SF-sLIG also achieved a lower PM penetration rate (or a higher efficiency) than the commercial filter. Both the dLIG filters had higher removal efficiencies and higher pressure drops but lower **QFs** than their sLIG counterparts.

The performance of DF-dLIG was not only superior to that of the fiberglass filter but also comparable to those of other novel filters. According to Wang et al., the removal efficiency of their novel electret filter could be as high as 99.97% against sodium chloride (NaCl) aerosol particles of size 0.3–0.5 μm [8]. Khalid et al. developed a transparent air filter and reported an efficiency as high as 99.95% against PM_2.5_, which decreased to 90.6% when the PM_2.5_ concentration rose above 708 μg/m^3^ [9]. Liu’s transparent filter achieved a 98.11% PM_2.5_ removal efficiency with ~70% light transmittance [10]. The conductive nanofiber membrane filters developed by Lee et al. reached a PM_2.5_ removal efficiency as high as 90% but had a minimum pressure drop of greater than 600 Pa at a flow rate of only 3.0 L/min [33]. 

### 3.4. Washability of LIG Filters 

The dLIG filter developed in this study exhibited outstanding washability with tap water due to its excellent water compatibility and mechanical durability. In contrast, the sLIG filter quickly disintegrated into separate LIG flakes in water. This dissimilar washability was dramatically demonstrated by cleaning sLIG and dLIG filters in 85 mL of tap water (mineral compositions: 20–100 mg/L, organic carbons compositions: ≤1 mg/L, turbidity: ≤0.3 NTU) [34] using a bath sonicator (UCP-02, ultrasonic power: 100 W, Jeio Tech INC., Seoul, Korea) for 30 s. 

**Figure 6:**
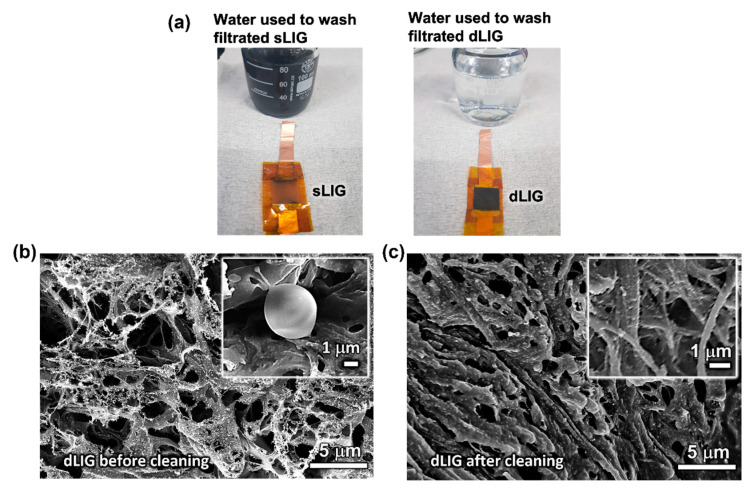
(**a**) sLIG filter and dLIG filter after cleaning with water, (**b**) Top-down view of dLIG filter after filtration, and (**c**) Top-down view of dLIG filter after washing captured via FE-SEM.

Figure 6a illustrates digital photographs of the cleaned sLIG and dLIG filters and the resultant cleaning water bottles. The LIG in the sLIG sample was quickly removed from the PI substrate and dispersed in water. In contrast, the dLIG sample retained its structure, and the amount of LIG flakes separated from the dLIG filter after cleaning was minimal in the cleaning water bottle. This result indicates the much stronger adhesion of the dLIG to the underlying PI and cohesion between LIG flakes in dLIG compared to those in the sLIG—a finding also supported by the SEM images of the sLIG and dLIG morphologies in the previous section. This stronger adhesion was possibly caused by the impregnation of PAA into the LIG mesh during densification. The spin coated PAA was situated in the porous LIG mesh and collapsed the fibrous LIG structures, binding them together with its high viscosity. When the coated LIG was irradiated, additional LIG appeared where PAA had impregnated the LIG mesh, resulting in much stronger bonding between the LIG flakes and a much denser porous LIG structure. 

The Coulombic force, used by the LIG filters to collect PM, dissipated when the filters were removed from the circuit. Consequently, the PM remained loosely bound to the surface of LIG. This mechanism allowed the PM to be removed quickly from the LIG filter by ultrasonication with just tap water. The use of an aggressive chemical solution was not needed to clean the dLIG filters. Consequently, dLIG filters are more easily and accessibly cleaned and retain their function after several usage and washing cycles. 

Figure 6b,c illustrate the FE-SEM images of the surface of a DF-dLIG filter captured after its first use in a filtration experiment (Figure 6b) and its subsequent cleaning (Figure 6c), respectively. After the first use during filtration, many PM particle agglomerates were observed on the surface of the DF-dLIG. The inset of Figure 6b illustrates a high magnification image of the PM collected in the LIG surface. Figure 6c reveals that agglomerates of PM could not be detected on the LIG surface after cleaning the used DF-dLIG by ultrasonication in tap water. The changes in the LIG morphology were possibly due to the wet cleaning process and subsequent drying. However, the structural integrity of LIG was maintained, suggesting the excellent washability and reusability of the DF-dLIG. 

**Figure 7:**
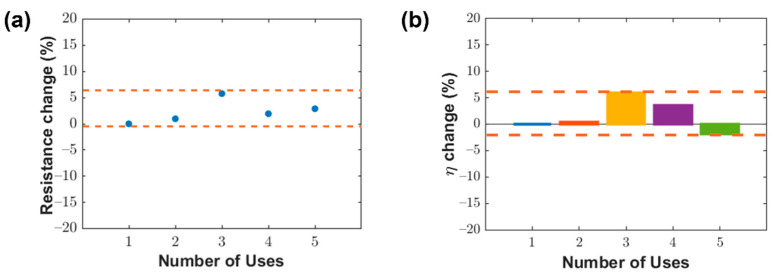
(**a**) Electrical resistance changes with repeated washing for DF-dLIG filter. (**b**) PM_2.5_ removal performance changes with repeated washing for DF-dLIG filter.

The effect of repeated water cleaning on DF-dLIG properties was also investigated. Likewise, a DF-dLIG filter was cleaned using a bath ultrasonicator multiple times. After each cleaning, the filter was dried on a hotplate at 65 °C for 15 min, with its resistance and filtration performance evaluated as described in Section 3.3. Figure 7a illustrates the relative changes in the resistance of the filter at its initial use and after four consecutive cleaning treatments. The relative resistance changes were less than 7%, confirming the robust characteristics of the DF-dLIG. Moreover, the filtration efficiency of the DF-dLIG filter remained at a high level after all five filtration and wash cycles (>99%), losing, at most, only a 2% filtration efficiency compared to the first use (Figure 7b). The efficiency changes in Figure 7b were obtained by averaging the data for the last 30 s of the entire 600 s filtration experiment.

The reusability of the DF-dLIG filter is competitive with those of other novel washable filters reported in the literature. The polybenzimidazole nanofiber filter developed by Lee et al. was washed in DI water and an organic cleaning solution [35]. Although the filter did not show apparent damage from the cleaning, its PM removal efficiency decreased after washing by approximately 6 and 3% for inorganic and organic PM, respectively, in the third use cycle. Li et al. developed a fibrous meltblown membrane filter using a zeolitic imidazolate framework-8/polypropylene–polycarbonate that was washable in DI water [36]. However, its filtration efficiency was considerably reduced by ~10% after five filtration-wash-dry cycles. Jeong et al. demonstrated a conductive Ag nanowire filter that could be cleaned in a mixture of ethanol, deionized (DI) water, and ethylene glycol [27]. A stable PM_2.5_ removal efficiency was observed after multiple reuse cycles, but a noticeable increase in resistance (up to ~45%) was observed.

## 4. Conclusions

We fabricated different LIG samples for PM filtration by irradiating a thin polyimide sheet with a CO_2_ laser. The LIG filters, especially DF-dLIG, feature high filtration efficiencies, minimal pressure drops, compact size, excellent durability, and high manufacturability. Compared to previously developed fibrous PM filters, which primarily use the dielectrophoretic forces of polar polymer nanofibers, the LIG filters enabled highly efficient PM collection by exploiting strong Coulombic forces induced when a bias voltage was applied to the filter. The DF-dLIG filter had a very high PM_2.5_ filtration efficiency (99.86%), while exhibiting a pressure drop of only ~19% of that of a conventional fiberglass filter. Moreover, the DF-dLIG filter demonstrated exceptional durability, sufficient for reuse after cleaning with water via ultrasonication.

## Figures and Tables

**Table 1:** Filtration performance summaries for different filter types.

Sample	E (%)	∆P (Pa)	QF (Pa-1)
SF-sLIG	96.82	278	0.0124
DF-sLIG	98.87	157	0.0285
SF-dLIG	98.01	366	0.0107
DF-dLIG	99.86	255	0.00771
Commercial	97.47	3764	0.00097

E: PM removal efficiency; ∆P: pressure drop; QF: quality factor.

## Data Availability

The data presented in this study are available in the article or supplementary material.

## Supplementary Materials

The following are available online at https://www.mdpi.com/article/10.3390/ma14195551/s1, Figure S1. Composition of generated PM (after dilution), Figure S2: Collection efficiency of different filters for different PM sizes, Figure S3: Digital image of the air filtration setup.
