# Supplementary material for: High-Performance Washable PM2.5 Filter Fabricated with Laser-Induced Graphene
Source: Materials (Basel). 2021 Sep 24;14(19):5551. doi: 10.3390/ma14195551 (PMC8509409; doi:10.3390/ma14195551)
Supplement: Supplementary file 1 [file materials-14-05551-s001.zip › materials-1377261-supplementary.pdf]

materials

MDPI

Supplementary Material

High-Performance Washable $PM_{2.5}$ Filter Fabricated with Laser-Induced Graphene

Anh-Phan Nguyen [1], Won-Kyu Kang [2], Jungbae Lee [2] and Jung-Bin In [1,2,*]

[1] Department of Intelligent Energy and Industry, Chung-Ang University, Seoul 06974, Korea; anhpn@cau.ac.kr
[2] Soft Energy Systems and Laser Applications Laboratory, School of Mechanical Engineering, Chung-Ang University, Seoul 06974, Korea; zk273rvlb@cau.ac.kr (W.-K.K.); skzzjd@cau.ac.kr (J.L.)
* Correspondence: jbin@cau.ac.kr

Citation: Nguyen, A.-P.; Kang, W.-K.; Lee, J.; In, J.-B. High-Performance Washable $PM_{2.5}$ Filter Fabricated with Laser-Induced Graphene. *Materials* **2021**, *14*, 5551. https://doi.org/10.3390/ma14195551

Academic Editor: Wiesław Stręk

Received: 27 August 2021
Accepted: 21 September 2021
Published: 24 September 2021

Publisher's Note: MDPI stays neutral with regard to jurisdictional claims in published maps and institutional affiliations.

Copyright: © 2021 by the authors. Submitted for possible open access publication under the terms and conditions of the Creative Commons Attribution (CC BY) license (http://creativecommons.org/licenses/by/4.0/).

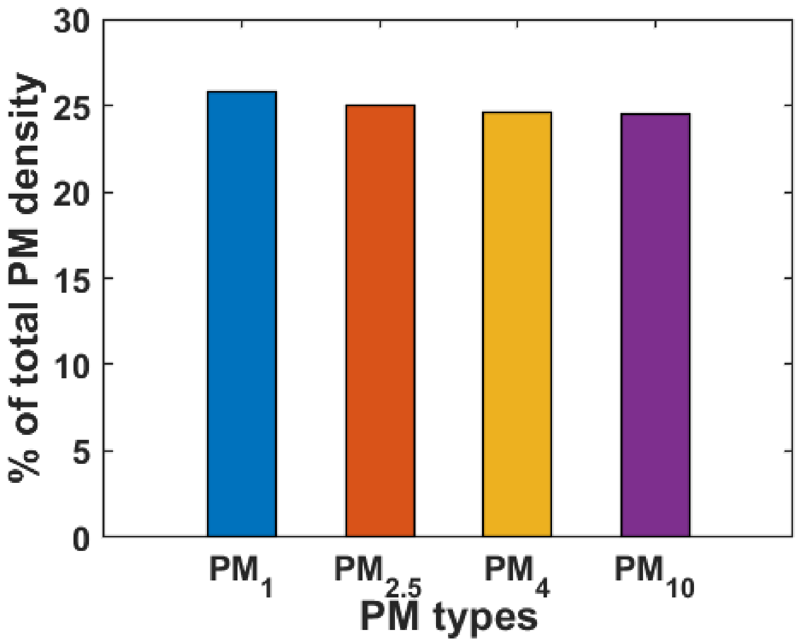

Figure S1. Composition of generated PM (after dilution).

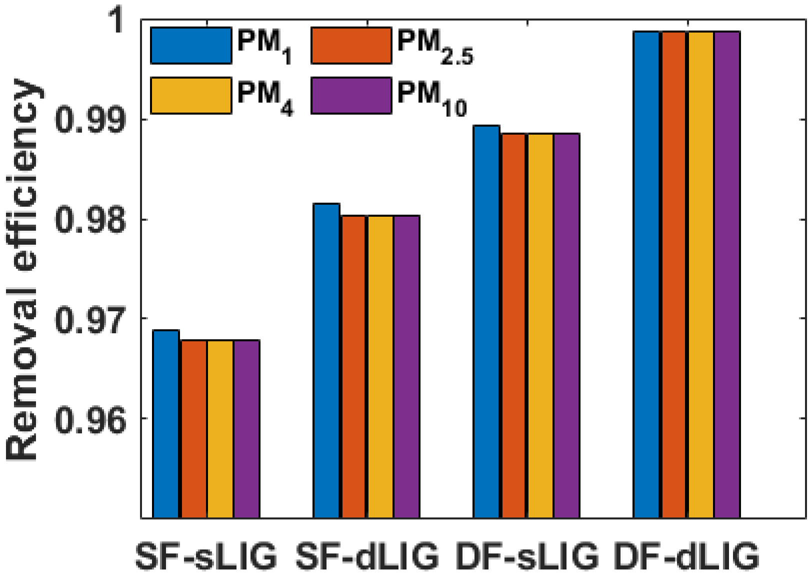

Figure S2. Collection efficiency of different filters for different PM sizes, with 12 V bias voltage.

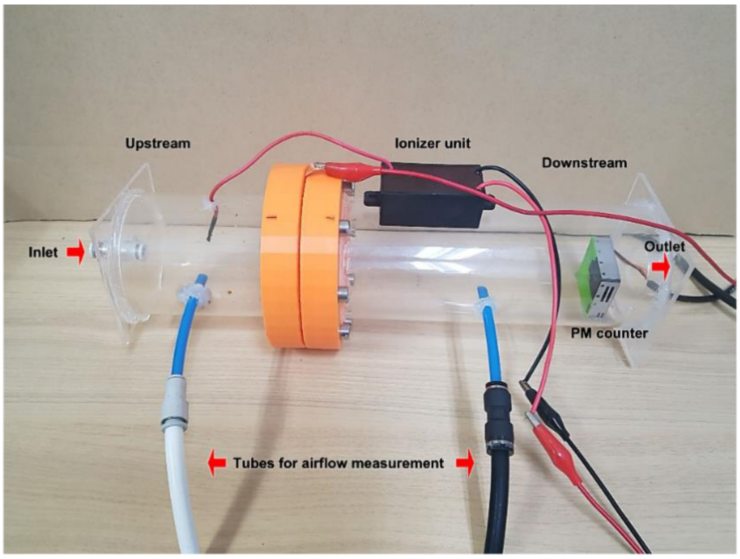

Figure S3. Digital image of the air filtration setup.
